# Draft Genome Sequence of *Bacillus aryabhattai* Strain PHB10, a Poly(3-Hydroxybutyrate)-Accumulating Bacterium Isolated from Domestic Sewerage

**DOI:** 10.1128/genomeA.01072-17

**Published:** 2017-10-12

**Authors:** Aneesh Balakrishna Pillai, Arjun Jaya Kumar, Kavitha Thulasi, Dinesh Reghunathan, Manoj Prasannakumar, Harikrishnan Kumarapillai

**Affiliations:** a Environmental Biology Laboratory, Rajiv Gandhi Centre for Biotechnology, Thiruvananthapuram, Kerala, India; b University of Kerala, Thiruvananthapuram, Kerala, India; c Genomics Core Facility, Rajiv Gandhi Centre for Biotechnology, Thiruvananthapuram, Kerala, India

## Abstract

*Bacillus aryabhattai* PHB10 is a poly(3-hydroxybutyrate) (PHB)-accumulating bacterium isolated from domestic sewerage. Here, we report the 4.19-Mb draft genome sequence, with 4,050 protein-coding genes and a G+C content of 37.5%. This sequence will be helpful in the study of the high-level PHB accumulation mechanism of the strain.

## GENOME ANNOUNCEMENT

*Bacillus aryabhattai* PHB10 was isolated from the environment as part of the screening of environmental bacterial strains for biopolymer production. The strain efficiently valorizes cheap raw materials by converting them to poly(3-hydroxybutyrate) (PHB) (1), which is a biodegradable and biocompatible plastic material of great commercial importance (2). In the present study, we investigated the whole-genome sequence of strain PHB10 for novel enzymes involved in PHB biosynthetic pathways. A typical PHB biosynthetic gene cluster with a class IV polyhydroxyalkanoate (PHA) synthase, reported previously in *Bacillus megaterium*, was identified in this strain (3, 4).

Genomic DNA was isolated from strain PHB10 using a Wizard genomic DNA purification kit (Promega, WI) according to the manufacturer’s instructions. A barcoded genomic library was generated using an Ion PGM library preparation kit and loaded into a 318 v2 chip (Life Technologies, Inc., CA). Sequencing was conducted in the Ion Torrent PGM platform and yielded a total of 3,003,051 reads with 60× genome coverage. The reads were trimmed and assembled into 72 contigs (*N*_50_, 271,936 bp) using SPAdes algorithm v 3.0.1 (5). The longest contig assembled was 645,097 bp. The total sequence length was 4,193,880 bp and showed a G+C content of 37.5%. The genome annotation was performed using NCBI Prokaryotic Genome Annotation Pipeline (PGAP) (6) and analyzed using the Rapid Annotations using Subsystems Technology (RAST) server (7).

The genome annotation identified 4,279 genes, 4,050 coding genes, 115 pseudogenes, 25 rRNAs, 84 tRNAs, and 5 noncoding RNAs (ncRNAs). The polyhydroxyalkanoic acid biosynthesis gene cluster was identified with the genes *phaP* (polyhydroxyalkanoic acid inclusion protein), *phaQ* (poly-beta-hydroxybutyrate-responsive repressor), *phaR* (polyhydroxyalkanoic acid synthase subunit R), *phaB* (acetoacetyl CoA reductase), and *phaC* [poly(R)-hydroxyalkanoic acid synthase subunit C]. A detailed comparison of the PHA gene cluster of *B. aryabhattai* PHB10 with other related genomes will be discussed elsewhere.

### Accession number(s).

The draft genome sequence can be accessed under GenBank accession number NOXE00000000. The version described in this paper is the first version, NOXE01000000.
